# The relationship between depression, healthy lifestyle behaviors and internet addiction: a cross-sectional study of the athlete university students in Turkey

**DOI:** 10.3389/fpsyt.2023.1222931

**Published:** 2023-07-07

**Authors:** Demet Öztürk Çelik, Meryem Öztürk Haney

**Affiliations:** ^1^Department of Physical Education and Sports Teaching, Osmaniye Korkut Ata University, Osmaniye, Türkiye; ^2^Faculty of Nursing, Dokuz Eylul University, İzmir, Türkiye

**Keywords:** athlete students, depression, healthy lifestyle behaviors, internet addiction, Turkey

## Abstract

**Introduction:**

Mental health research exists for university students in the areas of prevalence and association of depression, internet addiction and healthy lifestyle behaviors. However, the studies examining prevalence rates and these relationships in athlete university students are needed. This study aimed to evaluate the relationship between depression, healthy lifestyle behaviors and internet addiction among athlete university students in Turkey.

**Methods:**

A cross-sectional design was conducted, measuring depression using the Center for Epidemiologic Studies Depression Scale, healthy lifestyle behaviors (HPLP-II), and internet addiction (YIAT-SF). Predictive factors associated with depressive symptoms were estimated using binary logistic regression.

**Results:**

Of the 501 participants, 61.3% were male, with a mean age of 21.45 years (SD: 3.19). Student-athletes were at risk of depressive symptoms (76.4%), internet addiction (34.4%) and had moderate healthy lifestyle behaviors (130.41 ± 22.93). No significant differences were found for age, smoking, time spent on social network, number of sports days, number of social networks use for depressive symptoms (*p* > 0.05), however significant differences were found for depressive symptoms by sex and living with family status (*p* < 0.05). Pathological internet addiction (OR: 12.74, 95% CI: 2.91–55.77) and low spiritual growth (OR: 0.854, 95% CI: 0.79–0.92) were found to be predictors of depressive symptoms within the athlete university students (*p* < 0.001).

**Conclusion:**

The athlete university students are at increased risk for depressive symptoms and internet addiction and decreased healthy lifestyle behaviors. It is essential to establish programs to improve the mental health of students in universities and to strengthen the psychological counseling services offered to students. These findings can assist universities in establishing effective measures to improve mental health outcomes.

## 1. Introduction

Depression, which is characterized by sadness, loss of interest, hopelessness, anxious feelings, and even thought of suicide, increases during the transition from adolescence to adulthood; accordingly, depression is more common among university students than in the general population (1). University students are exposed to stress not only in their daily lives but also due to their education and additional stressors to which they are exposed in campus life. University students are a vulnerable group because they are in their developmental period, making efforts to adapt to a new environment, and trying to meet academic expectations (2). Recent studies report that approximately one third of university students displayed depressive symptoms (1). Studies show that the depression rates of Turkish university students are higher than those in the general population (3); these rates are also higher than those of university students in several other countries (4).

Many studies have found that depression among university students is associated with their lifestyle behaviors. According to these studies, behaviors such as cigarette smoking, hookah smoking, alcohol use, less healthy lifestyle habits, poor sleep quality, and internet addiction are associated with depressive symptoms (5–9). In addition, interventions that improve lifestyle play a role in reducing students’ depressive symptoms (10). In recent years, internet use has increased significantly around the world and has become an indispensable part of life. According to Internet World Statistics, 67.9% of the global population use the internet (11). This increase in internet use worldwide has led to the problem of internet addiction (12). Internet addiction, which is a subtype of problematic internet use, is the uncontrolled, excessive, and obsessive use of the internet causing physiological, social, and emotional problems in the individual (13). Meta-analysis studies show that the rate of internet addiction among university students is 31.5–34.5% in Africa (14). Studies in the literature indicate that 13–17.4% of university students in Turkey have internet addiction problems (15, 16); that internet addiction causes psychosocial problems such as depression, anxiety, insomnia, and low self-esteem in young people (17); and that the prevalence of depression increases with the severity of internet addiction (18).

Athlete university students are a subset of undergraduate students included in mental health statistics, and these students have additional stressors linked to mental health problems. For example, fear concerning a professional future, uncertainty regarding a professional career, vigorous exercise, and physical or somatic stress associated with training programs all affect the mental health of student athletes (19, 20). Previous studies report that participation in sports during university education facilitates positive mental health characteristics such as self-confidence, commitment, social support, and positive self-esteem (21). Student athletes who are confident and have high self-esteem are also able to make better connections with their environment and can combat depression and anxiety by utilizing social support systems to cope with stress more effectively (21, 22). Previous studies show that the internet addiction levels among athlete students are lower than those of other non-athlete students (23), and that internet addiction negatively affects happiness of athlete adolescents (24). The literature contains a limited number of studies on internet addiction in individuals who regularly engage in sports (23, 25); however, no study has yet revealed the relationship between internet addiction, healthy lifestyle behaviors, and depression in student athletes. Therefore, the primary aim of this study is to determine the prevalence of depression and the relationship between depression and healthy lifestyle behaviors and internet addiction in athlete university students in Turkey. The second aim of this study is to reveal those factors affecting depression in athlete university students in Turkey.

## 2. Materials and methods

### 2.1. Participants

This cross-sectional study was conducted in the faculty of physical education and sports departments of three state universities in Turkey between February and April 2022. Data were collected with online survey due to SARS-CoV-2 through the social media in order to minimize face-to-face contact. In February, a list of all undergraduate students continuing their education in the physical education and sports departments of the three universities was made and an informed consent form and a link to the measurement tools were sent to all undergraduate athlete students via email.

The sample comprised a total of 501 undergraduate athlete students. No sample selection method was used and all students who volunteered to participate in the study were included in the study sample. Out of a total of 1,480 eligible students, 34% were included in this study. Permission was obtained from the ethics committee of the Osmaniye Korkut Ata University (Date: 17.02.2022, Decision no: 2022/3/3) to which the first author of this study was affiliated. Written consent was obtained from the students who agreed to participate in the present study.

### 2.2. Instruments

#### 2.2.1. Demographic information form

This form, which was created by the researchers of the present study based on a review of the literature (12, 17), includes questions about the participants’ age, sex, whether they live with their family, smoking status, duration of social network use (hour/day), number of social networks they use, and frequency of physical activity (day/per week).

#### 2.2.2. Center for Epidemiologic Studies Depression Scale (CES-D)

The Center for Epidemiologic Studies Depression Scale (CES-D) is a self-report scale commonly used to monitor depressive symptoms in the general population (26). In this 20-item scale, participants are asked to evaluate the frequency of ideas and events over the past week and the answers are scored on a 4-point Likert-type scale (0 = rarely or none of the time, 3 = most of the time). Items 4, 8, 12, and 16 are scored inversely and total scale scores range from 0 to 60. A score of 0–15 indicates no depression, 16–20 indicates mild depression, 21–30 indicates moderate depression, and 30 and above indicates severe depression. The Cronbach’s alpha of the Turkish form of the scale was between 0.75 and 0.90 (27).

#### 2.2.3. Health Promoting Lifestyle Profile II (HPLP-II)

The health-related behaviors of students were assessed using the Health Promoting Lifestyle Profile II (HPLP-II) (28). The scale comprises 52 questions and six subscales: health responsibility (nine items), physical activity (eight items), nutrition (nine items), spiritual growth (nine items), interpersonal relations (nine items), and stress management (nine items). The frequency of participants’ practice of health promotion behaviors was assessed using a 4-point Likert-type scale (1 = never, 4 = routinely). All scale items were included as positive statements, with the lowest possible total scale score being 52 and the highest total score being 208. A high scale score indicates positive health promotion behaviors. The Cronbach’s alpha coefficient of the Turkish version of scale was 0.94 (29).

#### 2.2.4. Young’s Internet Addiction Test-Short Form (YIAT-SF)

The Young’s Internet Addiction Test-Short Form (YIAT-SF) is one of the most widely used measures of internet addiction (30, 31). The scale consists of 12-items that are rated on a 5-point Likert-type scale (1 = never, 5 = very often); the scale contains no inversely scored items. The minimum and maximum scores obtainable from the scale are 12 and 60, respectively. Scores below 31 indicate normal internet use, scores of 31–37 indicate problematic internet use, and scores above 37 indicate pathological internet use (31). The Cronbach’s alpha coefficient of the Turkish version of this scale was 0.91 for university students and 0.86 in adolescents (32).

### 2.3. Data analysis

Data were analyzed using SPSS 24.0. The demographic characteristics of the participants were presented using descriptive statistics (mean, standard deviation, and frequency). Normality of the data was evaluated using Kolmogorov–Smirnov test. Non-parametric tests were used in the study because the data were not normally distributed (*p* < 0.05). Comparison of categorical variables according to CES-D depression score was analyzed using the chi-square test. The relationship between the YIAT-SF and HPLP-II total and subscale scores and CES-D scores was assessed using Spearman correlation analysis. The comparison of YIAT-SF, HPLP-II totals and subscale scores with the CES-D depression scores was made using Mann–Whitney U test. The effect of demographic characteristics, internet addiction, and HPLP-II subscales on depressive symptoms was analyzed using binary logistic regression analysis. Values of *p* < 0.05 were considered statistically significant.

## 3. Results

### 3.1. Descriptive statistics

The students had a mean age of 21.45 years (SD: 3.19), and 61.3% of the students were male. The minimum and maximum age for male students were, respectively as 18, 38 and the minimum and maximum age for female students were, respectively as 18, 42. Of the 501 students, 55.1% were living with their families, 32.3% were smokers, 49.7% used social media 2–4 h a day, 39.9% did sports 3–4 days a week, and 49.5% used 3 or more social networks. The Cronbach’s alpha value was 0.89 for the CES-D, 0.94 for the HPLP-II, and 0.90 for the YIAT-SF. The mean CES-D score of the students was 24.32 ± 10.99. A total of 76.4% participants had depressive symptoms (14.4% mild, 32.5% moderate, 29.5% severe). The mean HPLP-II total score of the students was 130.41 ± 22.93 (ranging from 54 to 206). The mean item scores of HPLP-II subscales were as follows (from highest to lowest): spiritual growth 2.84 ± 0.55, interpersonal communication 2.77 ± 0.49, physical activity 2.48 ± 0.62, stress management 2.46 ± 0.51, nutrition 2.27 ± 0.52, and health responsibility 2.19 ± 0.57. The mean YIAT-SF score of the students was 28.03 ± 9.00. Of the students, 65.6% had no symptoms of internet addiction, 19.2% were addicted to internet at a problematic level, and 15.2% were addicted to internet at a pathological level. Detailed demographic information can be found in Table 1.

**TABLE 1:** Characteristics of the participants.

Characteristics		Total *n*	%
Total		501	100.0
Age (year) (mean ± SD)		21.45 ± 3.19	
	20 and below	228	45.5
	21 and over	273	54.5
Sex	Female	194	38.7
	Male	307	61.3
Living with family	Yes	276	55.1
	No	225	44.9
Smoking	Yes	162	32.3
	No	339	67.7
Time spent on social network (hour/day)	<2 h	125	25.0
	2–4 h	249	49.7
	≥4 h	127	25.3
Number of sports days (day/per week)	0	39	7.8
	1–2 d	174	34.7
	3–4 d	200	39.9
	≥5 d	88	17.6
Number of social networks used	≤2	253	50.5
	≥3	248	49.5
CES-D scores (mean ± SD)		24.32 ± 10.99	
	Normal	118	23.6
	Mild	72	14.4
	Moderate	163	32.5
	Severe	148	29.5
HPLP-II total score (mean ± SD)		130.41 ± 22.93	
Health responsibility (item mean ± SD)		2.19 ± 0.57	
Physical activity (item mean ± SD)		2.48 ± 0.62	
Nutrition (item mean ± SD)		2.27 ± 0.52	
Spiritual growth (item mean ± SD)		2.84 ± 0.55	
Interpersonal relationships (item mean ± SD)		2.77 ± 0.49	
Stress management (item mean ± SD)		2.46 ± 0.51	
YIAT-SF (mean ± SD)		28.03 ± 9.00	
	Normal	329	65.6
	Problematic	96	19.2
	Pathologic	76	15.2

### 3.2. Relationship between depression and demographic characteristics

Table 2 shows the relationship between the students’ demographic characteristics and their depression levels. The depression rate of female students (82%) was found to be significantly higher than that of male students (73%) (*p* < 0.05). Similarly, the depression rate of students who did not live with their families (81.3%) was found to be significantly higher than those who lived with their families (72.5%) (*p* < 0.05). No statistically significant difference was found between the students’ other demographic characteristics and their depression levels (*p* > 0.05).

**TABLE 2:** The relationship between students’ characteristics and depression.

		CES-D			
**Characteristics**		**No depression** **(*n* = 118)** ***n* (%)**	**Depression** **(*n* = 383)** ***n* (%)**	**χ ^2^**	***p***
Age	20 and below	52 (22.8)	176 (77.2)	0.129	0.719
	21 and over	66 (24.2)	207 (75.8)		
Sex	Female	35 (18.0)	159 (82.0)	5.341	0.021*
	Male	83 (27.0)	224 (73.0)		
Living with family	Yes	76 (27.5)	200 (72.5)	5.416	0.020*
	No	42 (18.7)	183 (81.3)		
Smoking	Yes	39 (24.1)	123 (75.9)	0.036	0.849
	No	79 (23.3)	260 (76.7)		
Time spent on social network (hour/day)	<2 h	38 (30.4)	87 (69.6)	6.002	0.050
	2–4 h	58 (23.3)	191 (76.7)		
	≥4 h	22 (17.3)	105 (82.7)		
Number of sports days (day/per week)	0	5 (12.8)	34 (87.2)	5.285	0.152
	1–2 d	38 (21.8)	136 (78.2)		
	3–4 d	48 (24.0)	152 (76.0)		
	≥5 d	27 (30.7)	61 (69.3)		
Number of social networks used	≤2	62 (24.5)	191 (75.5)	0.258	0.612
	≥3	56 (22.6)	192 (77.4)		

*p < 0.05, χ^2^ = Chi-square test.

### 3.3. Relationship between depression and healthy lifestyle behaviors and internet addiction

Table 3 shows the relationship between the students’ depression and HPLP-II total, subscale, and YIAT-SF scores. The total mean scores of HPLP-II, physical activity, spiritual growth, interpersonal relationships, stress management subscale scores of the participants with depression were found to be significantly lower than those without depression (*p* < 0.001). The mean YIAT-SF score of the participants with depression (29.67 ± 9.09) was significantly higher than were the scores of those without depression (22.72 ± 6.31) (*p* < 0.001). Spearman correlation analysis showed a significant positive correlation between CES-D and YIAT-SF (*r* = 0.445) and a significant negative correlation between CES-D and total HPLP-II and its subscales scores (physical activity, nutrition, spiritual growth, interpersonal relationships, stress management; *r* = −0.214, *r* = −0.149, *r* = −0.089, *r* = −0.35, *r* = −0.250, and *r* = −0.208, respectively) (*p* < 0.05, *p* < 0.001).

**TABLE 3:** The relationship between CES-D scores and subfactors and total scores on the HPLP-II and YIAT-SF.

	Spearman correlation with CES-D	No depression (mean ± SD)	Depression (mean ± SD)	*U*	*p*
HPLP II total score	−0.214**	139.20 ± 24.32	127.70 ± 21.82	4.869	<0.001**
Health responsibility	0.000	19.77 ± 5.54	19.74 ± 5.02	0.066	0.948
Physical activity	−0.149**	21.50 ± 5.32	19.33 ± 4.82	3.944	<0.001**
Nutrition	−0.089*	21.21 ± 5.10	20.27 ± 4.63	1.875	0.061
Spiritual growth	−0.351**	28.75 ± 4.97	24.69 ± 4.54	8.296	<0.001**
Interpersonal relationship	−0.250**	26.94 ± 4.65	24.38 ± 4.26	5.568	<0.001**
Stress management	−0.208**	21.01 ± 4.40	19.27 ± 3.99	4.044	<0.001**
YIAT-SF total score	0.445**	22.72 ± 6.31	29.67 ± 9.09	−9.337	<0.001**

*p < 0.05, **p < 0.001, U: Mann–Whitney U test.

### 3.4. Predictors of depression

Binary logistic regression analysis was applied by taking depressive symptoms as the dependent variable and the demographic characteristics, YIAT-SF, and HPLP-II subscales as independent variables (Table 4). Pathological internet addiction (OR = 12.74, 95% CI: 2.91–55.77) and low spiritual growth (OR = 0.854, 95% CI: 0.79–0.92) were found to be significantly associated (*p* < 0.001) with depressive symptoms.

**TABLE 4:** Results of logistic regression analysis, factors associated with depression.

Characteristics	*B*	Wald	OR	95% CI	*p*
Age	−0.020	0.300	0.981	0.914–1.052	0.584
Sex (male, ref.)	0.298	1.200	1.347	0.791–2.294	0.273
Living with family (yes, ref.)	0.496	3.763	1.643	0.995–2.713	0.052
Smoking (no, ref.)	−0.297	1.141	0.743	0.431–1.282	0.286
Time spent on social network (<2 h, ref.)					
2–4 h	0.093	0.096	1.097	0.611–1.971	0.757
≥4 h	0.399	1.169	1.491	0.723–3.074	0.280
Number of sports days (≥5 d, ref.)					
0	0.110	0.026	1.116	0.295–4.224	0.871
1–2 d	−0.222	0.273	0.801	0.349–1.839	0.601
3–4 d	−0.140	0.154	0.870	0.433–1.747	0.695
Number of social networks used (≤2, ref.)	0.001	0.000	1.001	0.601–1.642	0.997
Internet addiction (normal, ref.)					
Problematic	0.797	5.082	2.220	1.110–4.441	0.024
Pathologic	2.545	11.439	12.74	2.91–55.77	<0.001*
HPLP-II					
Health responsibility	0.097	7.056	1.101	1.026–1.183	0.008
Physical activity	−0.061	2.369	0.941	0.871–1.017	0.124
Nutrition	−0.002	0.002	0.998	0.924–1.078	0.960
Spiritual growth	−0.158	16.308	0.854	0.79–0.92	<0.001*
Interpersonal relationships	−0.037	0.884	0.963	0.891–1.041	0.347
Stress management	−0.024	0.260	0.976	0.891–1.070	0.610
Constant	6.035	28.924	417.607		<0.001*

*p < 0.001, OR, odds ratio; CI, confident interval.

## 4. Discussion

This study examined the relationship between depressive symptoms, healthy lifestyle behaviors, and internet addiction in Turkish athletic university students. The study results revealed that the prevalence of depressive symptoms was common (76.4%) among these students. In recent years, the prevalence of depression among university students has gradually increased (1). According to a meta-analysis study, in the literature the prevalence of depression among university students was 25% (33). Studies conducted with Turkish university students reported the prevalence of depression as 32.8–73.6% (34–36). A study comparing depression rates among university students in nine countries found that students in Turkey had the highest risk of depression and anxiety (4). A systematic review study examining the prevalence of depression among student athletes revealed that rates of depression among this population varied between 3.7 and 39% (37). A study conducted in China showed that the rate of depression among athlete students was 49.1% (21). The results of the present study are similar to those related studies from the literature and confirm that depression is an important health problem for student athletes in Turkey. A study associated high levels of depressive symptoms in Turkish athlete students with fear of professional future, uncertainty concerning professional career, and physical or somatic stress associated with severe exercise training programs (19). Depressive symptoms in athlete students can reduce their performance (38) and lead to injuries (39) and school dropout (40).

The results of the present study show that female students were also at risk for depression. A systematic review meta-analysis study found that female gender consistently increased the risk of mental health problems (33). In studies conducted with general university students and athlete university students in Turkey, female gender was identified as at risk for depression (36, 41). Wolanin et al. (42) stated that the risk of depression symptoms for the girls was 1.844 times higher than was that for the boys in athlete university students. Some studies in the literature suggest that social relationship factors were a possible explanation for gender differences in depression (43). A longitudinal study conducted with university students reported that both social isolation and loneliness were associated with increased depression symptoms in female university students; however, the study found that only social isolation was associated with increased depression symptoms in male university students (44). According to this result, gender differences in depressive symptoms were partly attributed to women’s experiences of more negative consequences of loneliness as compared to that of men’s experiences. The results of the current study confirm those in the literature and show that female students are at greater risk of experiencing depressive symptoms compared with male students.

In the present study, students who did not live with their parents had higher depression scores. Studies in the literature report that students living on campus/in a student hostel have more depression symptoms than those living with their parents (45, 46) and that being away from home may increase risks such as lack of social support and poor economic status. In addition, some studies have reported that moving away from home leads to stress (46, 47). The present study showed that students who lived away from their families had higher levels of depression symptoms.

The results of this study showed that 49.7% of athlete students used the internet for 2–4 h a day and approximately one third of all students had different levels of internet addiction. Previous studies in the literature conducted in Turkey reported that internet addiction among athlete university students was 26.4% (48), that internet addiction among non-athlete university students was 7.5%, and that the rate of potential addiction among these students was 34.4% (49). According to a meta-analysis study in the literature, internet addiction among young adults is prevalent internationally and internet addiction is increasing among new generations (50). The same study drew attention to the role of factors such as increased individuality, lower socialization, and acculturation in internet addiction. In the current study, participants’ internet addiction and depression scores were positively correlated, and the regression model showed that the risk of pathological internet addiction was 12.74 times higher among those with depression symptoms. The results of the current study are consistent with those of previous studies conducted with different student groups in Turkey. These studies reported that internet addiction was associated with high social anxiety, low self-esteem, and depression symptoms in medical faculty students (51), unhappiness in athlete high school students (24), and anger expression in athlete university students (52). According to the social-emotional model, negative emotions such as depression, anxiety, stress, and tendency to commit suicide also affect internet addiction (53). Internet addiction has also been found to be associated with a negative coping behavior of avoidance (54). The results of the present study confirmed that internet addiction in athlete university student was a risk factor for psychosocial problems such as depression.

The findings of the current study revealed that the HPLP-II total score of the athlete university students was moderate (130.41 ± 22.93), the highest healthy lifestyle behaviors were spiritual growth and interpersonal relationships, and the lowest behaviors were health responsibility and nutrition. Previous studies in the literature show that Turkish athlete university students had moderate HPLP-II scores, that the highest scores were spiritual growth/self-actualization, interpersonal relationships, and the lowest scores were physical activity, stress management, and nutrition (55, 56). In addition, this study also revealed a low-level negative correlation between HPLP-II total, physical activity, spiritual growth, interpersonal relationships, stress management scores, and depression scores of the participants. The results of present study are consistent with those of a study showing that healthy life behaviors are associated with depression, although the scale used to measure depression was different (57). The regression model showed that low spiritual growth posed 0.854 times more risk for depression symptoms. Inadequate self-actualization behaviors in university students are a risk factor for depression (12, 57). Spiritual growth refers to the individual’s search for meaning in life, a sense of belonging, the ability to choose freely among options, and maximizing the potential of healthy life through studies toward goals in life (29). Accordingly, it can be argued that because of the negativities in students’ efforts to find meaning in life and their efforts to attain belonging and personal goals in life, their general mental health status is negatively affected, especially as the severity of depressive symptoms increases.

Although this study is one of the few studies that evaluated the relationship between depression, healthy lifestyle behaviors and internet addiction among Turkish athlete university students, the study is not free from limitations and the findings should be discussed in line with these limitations. Firstly, even though the data of this study were obtained from athlete students of three universities in Turkey, the study sample represents a convenient sample of sports university students. Therefore, the study findings cannot be generalized to all athletic students. Future research should include a more representative sample. Secondly, the study sample was predominantly male students. Findings are limited to the sample of the study. Therefore, the obtained results may not be fully generalizable. Thirdly, due to the cross-sectional design of the study, it is not possible to establish a causal relationship between depression, healthy lifestyle behaviors and internet addiction. For the future, prospective studies are recommended to confirm the causal relationships between depression, healthy lifestyle behaviors and internet addiction in sport university students. In addition, future studies that take the gender distribution as similar are more likely to explore gender differences.

## 5. Conclusion

To the best of our knowledge, this study provides the first estimation of the relationship between depression, healthy lifestyle behaviors and internet addiction among athletic university students in Turkey. Our findings indicate that approximately two-thirds of the athletic students had depressive symptoms, one-third had internet addiction, and their healthy lifestyle behaviors were insufficient. The findings emphasized that depressive symptoms were more common in female students and students who did not live with their families, and that pathological internet addiction and low spiritual growth were the predictives of depressive symptoms. While the findings of the study support the results regarding the prevalence of depressive symptoms among university students in Turkey and in other countries of the world, it provides a novel data in terms of the prevalence of depressive symptoms among athlete university students in Turkey. The results suggest that improving healthy lifestyle behaviors in these students may help reduce their depression symptoms. Athlete students who have depression have a higher risk of developing internet addiction. Therefore, it is very important for universities and authorities to develop effective strategies to reduce depressive feelings in female students and in students who live separately from their families as both these groups are at greater risk. Accordingly, it is recommended that programs to improve the mental health of students in universities are developed and that the psychological counseling services offered to athlete university students are strengthened.

## Data availability statement

The raw data supporting the conclusions of this article will be made available by the authors, without undue reservation.

## Ethics statement

The studies involving human participants were reviewed and approved by the Ethics Committee of the Osmaniye Korkut Ata University (protocol no: 2022/3/3 and date of approval 17.02.2022). The patients/participants provided their written informed consent to participate in this study.

## Author contributions

Both authors contributed to the study’s conception and design, performed the material preparation and analysis, wrote the first draft of the manuscript, commented on the previous versions of the manuscript, and contributed to the article and approved the submitted version.
